# Pyroglutamic Acidemia: An Underrecognized and Underdiagnosed Cause of High Anion Gap Metabolic Acidosis - A Case Report and Review of Literature

**DOI:** 10.7759/cureus.5229

**Published:** 2019-07-24

**Authors:** Sidish S Venkataraman, Rachel Regone, Hussam M Ammar, Rukma R Govindu

**Affiliations:** 1 Neurological Surgery, Wake Forest University Baptist Medical Center, Winston-Salem, USA; 2 Otolaryngology, Kelsey-Seybold Clinic, Cypress, USA; 3 Internal Medicine, Medstar Washington Hospital Center, Washington, USA; 4 Internal Medicine, The University of Texas Health Science Center at Houston, Houston, USA

**Keywords:** anion gap, pyroglutamic acidemia, metabolic acidosis, 5-oxoproline, mudpiles, gamma-glutamyl cycle

## Abstract

Pyroglutamic acidemia (oxoprolinemia) is an underrecognized cause of high anion gap acidosis resulting from derangement in the gamma-glutamyl cycle. Pyroglutamic acidemia is most commonly diagnosed in the pediatric population in patients with inherited autosomal recessive enzyme deficiencies. However, acquired pyroglutamic acidemia can present in the adult population. Patients often present with confusion, nausea, and vomiting as well as an elevated anion gap metabolic acidosis. This article describes a case of acquired pyroglutamic acidemia and emphasizes the need to consider this entity.

## Introduction

Pyroglutamic acidemia is a condition in which the body is unable to produce glutathione. Glutathione is generated in the small intestines, kidney, and liver by the gamma-glutamyl cycle. The gamma-glutamyl cycle is responsible for maximizing the absorption of amino acids and plays an important role in chemical detoxification [1]. Most cases of pyroglutamic acidemia are reported in the pediatric population and are due to hereditary deficiencies in key enzymes involved in this cycle [2]. This paper, however, will focus on acquired derangements in the gamma-glutamyl cycle leading to depleted glutathione stores. While the depletion of glutathione can occur in patients with sepsis [3], liver disease [4], renal insufficiency [5], and malnutrition [6], the most notorious causes are alcohol abuse [7] and paracetamol (acetaminophen) consumption [8]. Paracetamol depletes glutathione stores. When glutathione stores are depleted, the gamma-glutamyl cycle "backs up" leading to the conversion of substrates into gamma-glutamyl amino acid by the enzyme gamma-glutamyl cyclotransferase [9]. Elevated levels of gamma-glutamyl amino acid can then lead to chronic acidosis, central nervous system damage, and hemolytic anemia. Treatment involves discontinuation of causative medications and treating any underlying medical conditions [10]. 

As the level of gamma-glutamyl acid increases, the patient will become more and more acidotic. The "GOLD MARK" mnemonic has recently gained popularity (over the mnemonic "MUDPILES") because it includes pyroglutamic acidemia as a cause for acidosis [11]. This mnemonic offers a differential to consider in patients with an elevated anion-gap metabolic acidosis. The mnemonic stands for: ethylene or propylene glycol (G), oxoproline, i.e. pyroglutamic acid (O), L-lactate (L), D-lactate (D), methanol (M), aspirin (A), renal failure, i.e. uremia (R), and ketoacidosis (K) [12].

## Case presentation

A 56-year-old woman was brought to the Emergency Center (EC) due to the recent onset of altered mental status and a three-day history of nausea and vomiting. She had a history of hyperlipidemia, hypothyroidism, depression, asymptomatic hepatitis C, and osteoarthritis for which she was taking simvastatin, ezetimibe, levothyroxine, trazodone, sertraline, and Vicodin®. 

On initial exam, she appeared older than her stated age, malnourished, dehydrated, and confused. She was found to have an arterial pH of 7.19, pCO2of 13.8 mmHg, HCO3 of 5.3 mmol/L, and an anion gap of 38.5. Her aspartate transaminase (AST) was within normal limits at 52 U/L and her alanine transaminase (ALT) was very slightly elevated at 69 U/L. A work up to investigate the cause of high anion gap metabolic acidosis was pursued. There was no osmolar gap which excluded methanol, ethanol, and ingestion of any other exogenous chemical as a cause. Uremia was excluded based on normal renal function. The salicylate and acetaminophen levels were found to be subtherapeutic. Only trace ketones were present in the urine, eliminating starvation and diabetic or alcoholic ketoacidosis. Her lactic acid was mildly elevated (3.7 mmol/L) but was not felt to be high enough to account for the clinical picture. Although no cause had been discovered for the patient's condition, a sodium bicarbonate drip, which was administered briefly, had begun to correct the metabolic abnormalities, and the patient's mental status had begun to improve. It was discontinued shortly thereafter so as not to overcorrect into an alkalosis. In subsequent questioning, the patient denied consuming isoniazid, paraldehyde, or excessive iron. She did report consuming one to two glasses of wine every day and four tablets of Vicodin® every other day for the past three years.

As there was no readily identifiable cause of her acidosis, and given her risk factors of malnutrition, alcohol intake, and her chronic acetaminophen use, pyroglutamic acidemia was suspected. Subsequently, a urine sample was sent for organic acid analysis, which revealed a "large elevation" of pyroglutamate (5-oxoproline), thus confirming the diagnosis. Our patient's anion gap and acidosis resolved, and after a few days of treatment and counseling, she was discharged in stable condition and returned home. She has not had any subsequent similar admissions since that time. 

## Discussion

**Figure 1:**
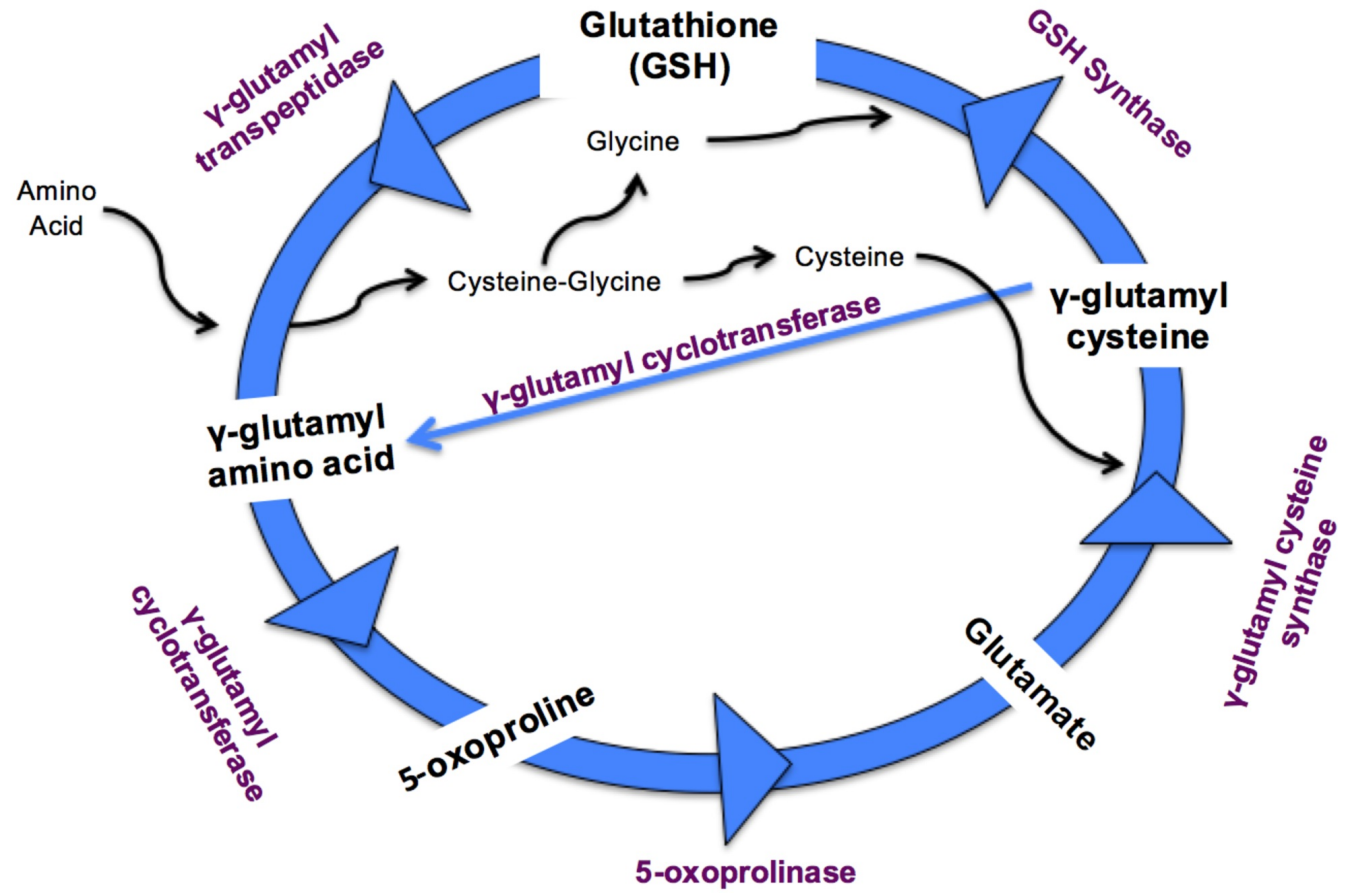
Gamma-Glutamyl Cycle Representation of the gamma-glutamyl cycle [14]. GSH - glutathione.

Glutathione is a tripeptide present in most mammalian cells and functions as a free radical scavenger, detoxifier of carcinogens and xenobiotics, and participates in neurotransmission, redox reactions, and the biosynthesis of DNA, proteins, and leukotrienes [13]. Five enzymes are involved in the metabolism of glutathione in the gamma-glutamyl cycle [13], see Figure 1. This cycle occurs primarily in three locations in the body: the small intestines, kidney, and liver [1]. The main function of the gamma-glutamyl cycle (also known as the Meister cycle) is to maximize the absorption of amino acids, especially from the small intestine and in the proximal tubules of the kidneys. However, in the liver, this cycle plays an important role in chemical detoxification [1].

Autosomal recessive hereditary deficiencies have been found in each of this cycle's necessary enzymes, with glutathione synthetase deficiency being the most common [13]. This deficiency is associated with 5-oxoprolinuria along with chronic acidosis, anemia, and central nervous system damage, and although rare, was one of the most commonly recognized causes of pyroglutamic metabolic acidosis [13] until 1989 when an acquired deficiency was first described in an adult [15]. The even rarer 5-oxoprolinase deficiency is also associated with 5-oxoprolinuria [13]. Both of these enzyme deficiencies are typically discovered during childhood and were therefore initially described in pediatric literature [2]. 

Since 1989, when the first case of acquired pyroglutamic metabolic acidosis was reported, numerous additional cases have been described. These cases have shown that although rarely diagnosed (if not rare in occurrence), pyroglutamic acid accumulation does occur in the adult population without a hereditary enzyme deficiency, typically presenting with a high anion gap metabolic acidosis along with mental status changes [9]. 

It is thought that the accumulation of pyroglutamic acid occurs as a result of depletion of glutathione stores, which removes the negative feedback and essentially “backs up” the cycle, leading to elevated levels of 5-oxoproline. This excess acid then exceeds compensatory mechanisms, leading to pyroglutamic metabolic acidosis [9]. Glutathione can be depleted through a variety of mechanisms. Acquired mechanisms include direct depletion of the glutathione stores and indirect depletion through transient inhibition of enzymes involved in the cycle, such as 5-oxoprolinase [9]. Since our patient was not identified in childhood and did not describe any symptoms prior to adulthood, an acquired deficiency due to the depletion of glutathione stores is the most likely cause that led to her condition.

**Figure 2:**
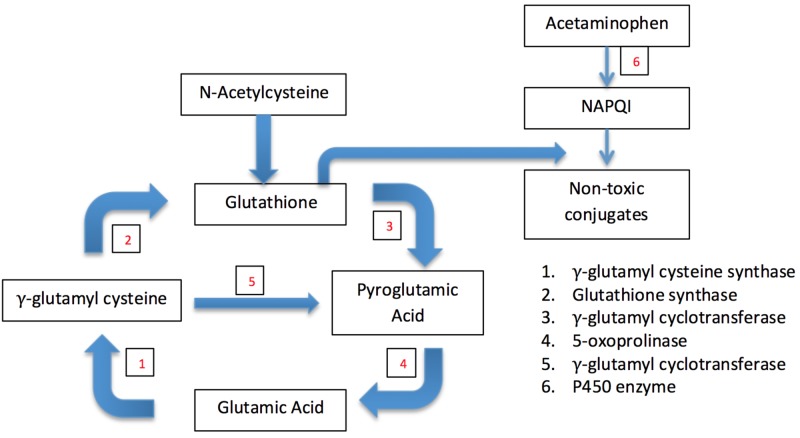
Paracetamol's Effect on the Gamma-Glutamyl Cycle The way in which acetaminophen affects the gamma-glutamyl cycle leading to 5-oxoprolinuria (pyroglutamic acid). Glutathione is needed to detoxify toxic metabolites of acetaminophen. With glutathione stores depleted, there is excess gamma-glutamylcysteine, which is converted to pyroglutamic acid by gamma-glutamyl cyclotransferase [4, 9, 14]. NAPQI (N-acetyl-p-benzoquinone imine) is a toxic byproduct generated during the metabolism of acetaminophen in the liver. The numbers in red correspond to the enzyme involved in that reaction.

Identifying this patient's unique risk factors for glutathione depletion remained a clinical question. Past reports of adults with 5-oxoprolinuria have described several potential contributing culprits of such a depletion: sepsis [3], liver disease [4], renal insufficiency [5], malnutrition [6], alcohol abuse [7], artificial feeds [16], and a vegetarian diet [17]. As well as, pregnancy, type 2 diabetes, and certain medications, including vigabatrin, monosodium glutamate, flucloxacillin, netilmicin, and most notoriously, paracetamol [10]. Female sex also appears to be a risk factor, as women have different levels in the activity of glutathione transferase and lower stores of glutathione than men [18]. Paracetamol (acetaminophen) plays a significant role through its direct depletion of glutathione stores in the liver, while vigabatrin and flucloxacillin work through a different mechanism by inhibiting the enzyme 5-oxoprolinase [10]. As shown in Figure 2, the depletion of glutathione would lead to a decrease in its negative feedback, producing an accumulation of gamma-glutamyl cysteine [9]. Normally this would, in turn, lead to increased glutathione through glutathione synthetase, but it is thought that glutathione synthetase may become rate-limiting or may even be inhibited by acetaminophen or a product of its metabolism [9]. The excess of gamma-glutamylcysteine through gamma-glutamyl cyclotransferase can then produce 5-oxoproline.

Any of the above factors alone are unlikely to produce a profound metabolic acidosis; however, a combination of factors could produce such a metabolic disturbance. This was likely the case in our patient, given her chronic acetaminophen ingestion (and thus depleted glutathione stores), alcohol use, and malnutrition, which culminated in her presentation with pyroglutamic acidemia.

When pyroglutamic acidemia is suspected, potentially causative medications should be stopped immediately, and contributing medical conditions such as sepsis or underlying renal failure should be addressed. Many cases have also reported treatments for the acidosis, which have included extracellular volume expansion using saline containing dextrose [19], bicarbonate, N-acetylcysteine, and even dialysis. Because of its ability to regenerate glutathione, N-acetylcysteine seems to be an obvious choice for treatment and appears to be the strategy most often employed according to the literature [20]. However, as of yet no trials have shown which treatment method and strategies for prevention are most effective. These need to be identified in order to develop appropriate guidelines for similar situations.

There are numerous reports which discuss similar scenarios, pointing to the fact that this is neither an uncommon problem nor solely a problem seen in pediatric patients, as was once thought. However, it is still under-recognized, as evidenced by our patient’s previous undiagnosed presentation. One potential cause for this condition becoming more prevalent and at the same time contributing to its under-recognition is the abundant and widely accepted use of acetaminophen. The drug is frequently prescribed, easily accessible over the counter, and is generally thought of as a safe medication. This condition should be suspected in any patient presenting with high anion gap metabolic acidosis who has a history of acetaminophen use either alone or in combination with a narcotic, such as Vicodin® [20]. There is a need to acknowledge and consistently incorporate pyroglutamic acid into the differential, not only in pediatric patients but also in the adult population.

## Conclusions

Pyroglutamic acidemia is an underrecognized cause of elevated anion gap metabolic acidosis. The objective of this case is to increase awareness that not just one factor alone but a combination of factors, such as chronic acetaminophen use, alcohol use, and malnutrition as seen in our patient, could lead to this type of acidemia. In these cases, all contributing factors should be identified and corrected, including discontinuation of potentially contributing medications and treatment of any underlying medical conditions. Failure to do this could lead to recurrences if not all factors are addressed. Continually more cases of pyroglutamic acidemia have been recognized, leading one to the conclusion that this cause of metabolic acidosis may not be as uncommon as initially thought.
